# Perspectives of psychiatric nurses on the stigmatization of mental healthcare in Ghana: a qualitative study

**DOI:** 10.3389/fpubh.2024.1423445

**Published:** 2024-10-25

**Authors:** David Kofi Mensah

**Affiliations:** Department of Anthropology, Northern Arizona University, Flagstaff, AZ, United States

**Keywords:** mental health, stigma, psychiatry, psychiatric nurses, Ghana

## Abstract

**Background:**

Stigma surrounding mental illness has been identified as a major bane of psychiatric care in many low- and middle-income countries. Mental illness stigma affects both the sufferer and their care providers, including families and psychiatric care providers. In Ghana, attention toward psychiatric care is limited. This article provides qualitative insights on interpersonal and structural stigma experiences of psychiatric nurses from the Ankaful Psychiatric Hospital, Ghana, and the impact of these stigmas on psychiatric care provision in Ghana. The article addresses relevant gap in the literature on the impact of mental illness stigma experiences among psychiatric nurses in low- and middle-income countries.

**Methods:**

Using a qualitative research approach, this study draws from the experiences and perspectives of stigma among psychiatric nurses from the Ankaful Psychiatric Hospital. Fourteen registered psychiatric nurses were recruited and interviewed using semi-structured interviews with open-ended questions. A thematic analysis approach was adopted to analyze the data. Coding and analysis were done in NVivo 12, aiding in the analysis of major themes and subthemes that emerged from the data. The study was conducted remotely due to the COVID-19 pandemic travel restrictions.

**Results:**

Themes identified include social and structural stigmas toward psychiatric nurses and the impact of these stigmas on mental healthcare in Ghana. While social stigma makes psychiatric nursing a difficult career choice for many, structural stigma—resulting from governmental neglect and lack of material resource provision—presents difficulties for nurses and other psychiatric care providers in the exercise of their care duties, which negatively impacts care provision for persons with mental health issues.

**Conclusion:**

The relevance of the experiences and perspectives of psychiatric nurses on mental illness stigma cannot be overlooked in the effort to promote mental illness advocacy, treatment seeking, and improve psychiatry. This article provides insights into the stigma experiences of psychiatric nurses and the impact of these stigmas on mental healthcare provision in Ghana. The study contributes to efforts to improve mental healthcare policymaking and advocacy.

## Introduction

Over the past two to three decades, advocacy and research toward mental illness and mental healthcare have gained attention globally. Stigma of mental illness and its impact on treatment seeking and care provision continue to gain central attention in mental health research. However, the focus has mostly been on people with mental health issues, but less on psychiatric care providers. According to Kohrt and Mendenhall (1), there is limited research on mental health service providers—psychiatric nurses, psychiatrists, mental health counselors and therapists, among others, and policymakers—advocacy groups, professional bodies, and governmental and non-governmental agencies involved in mental health policy enactments. These groups are essential players in bridging the mental health treatment gaps and the scaling up of mental healthcare provision, especially in low- and middle-income countries, which is one of the primary goals of the global mental health movement (2, 3). Research focus on these groups is indispensable to the advocacy and promotion of mental healthcare globally. This article focuses on interpersonal and structural stigma experiences of psychiatric nurses in Ankaful Psychiatric Hospital and their impact on mental healthcare in Ghana.

Psychiatric nurses are an essential body in the mental health service provider group and play a pivotal role in finding solutions to mental healthcare issues (4, 5). To ensure a robust improvement in mental healthcare service provision and implementation of policies, there is a need to involve service providers, especially psychiatric nurses. Omar et al. (6) identified that mental health research and policies in Sub-Saharan Africa rarely focus on psychiatric nurses. The conditions of service and social engagement of psychiatric nurses in their communities impact care provision (7, 8). Thus, there is a need for attention toward psychiatric nurses by Global Mental Health (GMH) researchers and advocates.

Literature on mental health shows that mental healthcare is neglected in many middle- and low-income countries with little or no budgetary allocation (9–13). This situation is not solely due to a lack of resources; beliefs and attitudes toward mental illness also play a significant role (14). Attention must be directed to the sociocultural factors that influence the decisions and approaches of policymakers and mental health professionals and their impact on mental healthcare. Social stigma has been identified as the leading sociocultural factor affecting mental health advocacy, policymaking, and healthcare provision (9, 15). Kapungwe et al. (16) assert that stigma is one of the social and behavioral factors affecting mental healthcare in Zambia, just as in any other Sub-Saharan African countries and low- and middle-income countries. A review of literature on mental health research in Sub-Saharan Africa suggests a gap in the literature on the views of various stakeholders, especially mental healthcare service providers and policymakers, on why mental health remains a low priority in Sub-Saharan African countries and what can be done (11, 17–19).

The Mental Health and Poverty Project (MHaPP) Research Programme Consortium’s research among four Sub-Saharan African countries—Ghana, South Africa, Zambia, and Uganda—has been one of the major mental health research projects in the sub-region to investigate views of stakeholders on mental healthcare (11). The study identified plethora of challenges and limitations facing mental healthcare in these four countries, which include inadequate human resources (12), poor mental healthcare systems, weak mental health policies (6), and suggests the need for integration of mental health into primary healthcare (20) and the involvement of service users in policymaking (18). In many Sub-Saharan African countries, the stigma attached to mental illness and other socioeconomic factors influences governmental decisions on mental health in annual budgetary allocation for healthcare systems, as identified by Omar et al. (6) in their study in South Africa. The MHaPP research project suggests there is low priority on mental health by both government officials and the public in Sub-Saharan African countries, resulting in a lack of recognition of mental health in governmental agenda-setting (11). Research identifies that psychiatric nurses, who spend most of their time with patients, are rarely involved in mental health policy discussions (6, 18, 19, 21).

Studies on mental illness stigma in Sub-Saharan Africa (SSA) show that the stigma of mental illness arises from the understanding of mental illness as a bad omen or possession by a bad spirit and the unpredictability of the mentally ill being aggressive (16, 22, 23), leading to fear of contagion and violence ascribed to persons with mental illness. There is a common societal belief or perception that a bad spirit can possess a person, infect them with mental illness, and make them unpredictable and dangerous, as well as a spiritual threat. Hence, the mentally ill are shunned, pushed away, and avoided (24–26). People with mental illness then face apprehension leading to fear and avoidance, as they are perceived as a source of physical and spiritual contamination. This does not only apply to persons with mental illness, as their families and healthcare providers face stigmatizing behaviors and attitudes due to their relationship with the mentally ill person.

This article looks at the impact of stigma toward psychiatric nurses on mental healthcare in Ghana. It provides qualitative insights into interpersonal and structural stigma experiences of psychiatric nurses from the Ankaful Psychiatric Hospital, Ghana. There is the need for more research on the experiences and perspectives of psychiatric care providers on the advocacy and promotion of mental healthcare using different research methodological approaches. The relevance of the experiences and perspectives of psychiatric nurses on mental illness stigma is pivotal in the effort to promote mental illness advocacy, treatment seeking, and improve psychiatry.

## Methods

### Study design

The study draws from the experiences and perspectives of psychiatric nurses from the Ankaful Psychiatric Hospital to explore the experiences of Ghanaian psychiatric nurses in the provision of mental healthcare in Ghana. Anthropological perspectives and methodologies, including semi-structured interviews and conversations, were used to explore these experiences and perspectives. These methods provide specific and in-depth information and engagement of research participants to understand their experiences and perceptions of the research topic (27). The main research questions include: (1) What are the experiences of psychiatric nurses in providing mental healthcare in Ghana? (2) What are the factors that impact mental healthcare delivery in Ghana? The study methods initially included interviews with patients, family members of patients, and healthcare providers (psychiatrists, psychologists, psychiatric nurses, and physician assistants) at the Ankaful Psychiatric Hospital, but due to the COVID-19 pandemic, the research was limited to remote interviews with psychiatric nurses. Nevertheless, the main research questions did not change; the study explored the same themes from the perspectives of only the psychiatric nurses. Open-ended, semi-structured interview questions were developed to answer the research questions and to achieve the objectives of the research. Interviews were conducted using both English and Twi languages.

### Study setting and participant

The study was conducted remotely with 14 registered mental health nurses from the Ankaful Psychiatric Hospital in Cape Coast, Ghana, one of three major psychiatric hospitals in Ghana. Aside from the psychiatric care services, the hospital has special clinics for epilepsy, alcoholic anonymous meetings and 12-step rehabilitation, a maternal care unit, a diabetes and hypertension clinic, and general medical outpatient services. The hospital was chosen due to its location and the number of communities and regions they serve in the country. The study specifically recruited only mental health nurses due to the COVID-19 pandemic travel restrictions in 2020, which limited the research to remote interviews and conversations with participants. Owing to the proximity of nurses to patients, the mental health nurses presented the best option in such a tight situation in soliciting mental illness care experiences from care providers at the hospital. Participants were between the ages of 25 and 35 years, Ghanaian born and raised, and from different regions and ethnic backgrounds.

### Sampling and data collection

Convenience and snowball sampling (28) were used to recruit participants for the study. Due to limitations presented by the COVID-19 pandemic, the study was internet-based, and participants were recruited based on referrals from previously interviewed nurses. Fourteen registered mental health nurses were recruited and interviewed using semi-structured interviews with open-ended questions to answer the research questions and to achieve the objectives of the research. Interviews were more of a conversation than ‘question and answer’ session. This approach offered participants the opportunity to express their feelings and perceptions about the issues being discussed. This piqued the interest of participants as they became more comfortable talking and expressing themselves without restriction. Interviews ranged between 50 min and 70 min, depending on how participants engaged with questions from the interview guide.

### Data analysis

Interviews were transcribed verbatim, a mixture of English and Twi languages. This helped to maintain the originality of the interviews and retain the original meanings of native words used to explain certain phenomena expressed by participants. To familiarize myself with the data, notes taken during interviews and interview transcripts were printed and manually coded based on the research questions. Further in-depth coding was done in NVivo 12, aiding in analyzing major themes and subthemes that emerged from the data. A codebook was generated to summarize and write code reports of emergent themes through description of each identified theme. A thematic analysis approach (28) was used to analyze the data in its final stage. Themes generated from the data were based on participants’ accounts of their experiences and perspectives on mental illness stigma and mental healthcare in Ghana from interviews. Themes were then reviewed and refined to help write the research results presented in this article.

## Results

### Interest in psychiatric nursing

Participants shared that some psychiatric nurses in Ghana had no initial interest in pursuing psychiatry but were placed in the sector when applying for nursing training colleges. They explained that because there is a centralized nursing admission system in Ghana, those who do not get admission into the general nursing schools may be given admission to the few psychiatric nursing training colleges in the country due to low applications to the psychiatric training colleges. One nurse explained that:

*Most of the time what happen is that when the sister schools, the general nursing training college, when people buy their form and they are full then they sometimes push it … yah they send the eh the left-over people’s form to the psychiatric hospital. So, people even come and without knowing that they are coming to do psychiatry until they go for their first orientation.*

Almost all participants did not have initial interests in becoming psychiatric nurses. Though most of the participants wanted to work in the healthcare sector, psychiatry was not part of their plans as it is unpopular in Ghana due to how people perceive mental illness and anything that is associated with it:

*Well, mental health nursing, honestly [in the] beginning I also had some kind of fear and then some stigma attached to the profession, honestly! We really had no idea what goes on in the mental hospitals and even the school (psychiatric nursing training college) we had less knowledge in them. So, growing up we all wanted to become nurses, but it was about either midwifery or general nursing […] but later I was advised by a friend that mental health is superb. It’s a specialty, so you are being regarded when you are seen as a psychiatric nurse.*

Nurses, however, asserted that people are also demotivated to pursue psychiatry due to the perception that psychiatric hospitals house ‘wild’ and ‘dangerous’ people who will harm you at any given opportunity. A nurse explained that “…people have made their mindset that mentally sick patients are harmful, when you do not protect yourself, they will hurt you.” They asserted that it is one aspect of the negative perception about psychiatry that discourages people to enter the field. One nurse shared that she “had that kind of fear that ei! this people they are mad! That’s what I was thinking, they are mad and they are on the road, how do we cater for them, so I wasn’t really interested.” One female participant who developed interest prior to her enrollment in the psychiatric training college explained that she had compassion for mentally ill people when she sees them on the streets and are being treated inhumanely, and thus, decided to become a psychiatric nurse to care for them:

*…as far as life is concerned, care must be taken or given to others as well and as such I developed […] passion for [mentally ill] people. For instance, when I see people walking by the streets. I have this one of my seniors who went to the university training school. So, upon interacting with him when I graduated from SHS, he told me about psychiatric nursing. I mean I was aspiring to be a nurse, so he explained all the categories of nursing to me and finally I chose that one […] I prefer to do that because that’s where I see my passion to be. So that’s how come I choose to be a psychiatric nurse.*

Almost all the participants had their perception and understanding of mental illness changed after getting into psychiatry. They have come to realize that mental illness and mental healthcare are not as they have perceived them and that it is societal perceptions about mental illness that have made the sector unattractive to people who want to pursue nursing or work in the mental healthcare sector. Participants asserted that there is a negative perception about mental illness and mental healthcare in Ghana, as one nurse professed, “I think when I entered the profession, I noticed that everything was not like the way I think.”

Participants were appreciative of the opportunity to pursue psychiatry because it has helped them to understand more about human complexities. They asserted that psychiatry has helped them to relate well with all manner of behaviors from people in their societies, as this nurse explained:

*…during the schooling, my second year first encounter with them on the ward I was like ei! this people are not like how we see them outside, they are a different people altogether and getting closer to them you get to understand how human beings are and then how to we are able to quickly relate with these ‘mad people’ as quickly than I think you’d have the chance of relating to other people we see them to be normal. …getting closer and then through the education I think the interest grew as the days went by.*

Another nurse affirmed that:

*it’s one of the best choices that I’ve made.*

### Social stigma toward mental health nurses

Mental healthcare workers face stigmatizing situations almost everywhere they find themselves. According to the nurses, mental illness stigma is a widespread phenomenon in Ghana that affects not only patients and their families but mental healthcare workers as well. Due to their close relations with mentally ill people, most people in the society do not see anything good in psychiatric nurses; hence, do not accord any respect to them, as in the narration of this nurse:

*Oh, I think Ghana they do not respect mental health nurses. Yes, that’s what I will say they do not regard it at all. They do not see it as part of eh the nursing. All they know is general nurses, midwife, community nurses but woyԑ [if you are a] mental health nurse ah, you are being stigmatized.*

Participants asserted that “normally it’s the general public that stigmatizes us kraa who are working there.” The nurses explained that they face stigma just like their patients from the society. Because of the beliefs and attitudes people have about mental illness and anything associated with it, they tend to stigmatize and disrespect mental healthcare workers and do not regard them as healthcare professionals as they do to other healthcare professionals such as primary healthcare doctors, nurses, and midwives. Participants explained that, at the mention of “I am a psychiatric nurse, people’s expression towards you changes, and they begin to question you on your job.” One nurse narrated that:

*I remember during the school days nu, me maame adamfo bi, me maame ka kyerɛ no sɛ me yɛ nurse. Me kɔ yɛ na ne ba no se ɔbɛyɛ nurse, na obisa me sɛ me kɔ school bɛn na me se me kɔ Psychiatric Nurses Training College, eei abɔdam foɔ nurse na wo bɛ yɛ na wobetumi, saa nursing yi, have you seen?* [I remember during my school days, my mother told one of her friends that I am a nurse, and she asked me which school I attend, and I said Psychiatric Nursing Training College, then she shouted, “mad people’s nurse, can you do it?”]

Participants explained that dealing with mental health stigma is sometimes an individual affair, but they mostly use their education to navigate through such times. They believe that the stigma is based on people’s perception about mental illness; thus, educating such people on mental illness helps to change their attitudes toward mental illness a bit. Unlike mental health patients who encounter difficulties integrating into society after recovery, the psychiatric nurses explained that they are able to stand stigma because of their education and understanding that it comes with their job, so they are “able to deal with stigma in a more professional way.” According to the psychiatric nurses, when they face stigmatizing situations and can talk to such people, they educate them about mental illness and their profession. One nurse explained that:

*Oh, you know, one thing to, when we got to the first year, we were taught about stigmatization. We were taught about stigmatization. That was the first thing that we were taught, so that motivates us a lot. So, whenever somebody says things like that to us, we in turn tend to educate them about mental health and in the long run I think they do appreciate it a lot. Because most of them they do not have much education about mental health in general and once they start saying those kinds of things and we tend to give them the education on mental health, then they begin to appreciate it and I think that helps a lot, yeah.*

### Inter-provider stigma

Participants explained that their colleague healthcare providers, especially general nurses, and midwives in the primary healthcare sectors stigmatize against them and their patients. The nurses asserted that the general nurses stigmatize and treat mental health nurses as if they do not belong to the same healthcare system in Ghana. Participants explained that they did not know about stigma from colleague healthcare providers till they got into the field, as in the narrative of this nurse:

*So, I entered the field because before I realized that there were a lot of stigmas not pertaining to the patients, but we the staff, we were even stigmatized. Even when we are on clinicals, vacation clinicals and you go to the general hospitals and they see you they are like [abɔdam] mad nurse, [abɔdam] nurse ahaa, you know. It’s like they stigmatize we the staff even together with the patients and some even go as far as saying that they hear that if you are working at the psychiatric hospital, a time will come, and you start behaving like the patients.*

Another nurse opined:

*…even your own other health workers that are supposed to be enlightened about mental illness, they are the people that will be stigmatizing you that you are behaving like your patient and comparing you to your clients and all sort of stuff.*

Participants asserted that due to stigma, they are sidelined in major nursing programs, and during meetings, they are hardly recognized or given the opportunity to talk. Recounting their encounters with general medical nurses and nursing students during school days, participants pointed out the segregation and disrespect they face during clinical, as in this narration:

*…when you go to the maternity wards na ɔmo bisa sɛ woyɛ nurse bɛn na wo ka sɛ wo yɛ psychiatric nurse a, gyai! [and they ask which type of nurse are you and you mention psychiatric nurse, stop!] all of a sudden na ɔmo asesa [they change] towards you asɛ wo nnim hwee [as if you do not know anything]. Psychiatric nurses deɛ mo yɛn hia mo wɔ ha, mo ba ha kraa [we do not need you here, why are you even here] like that kind of thing. But we learn what you guys are doing. We have psychiatric patients who get pregnant, and we take care of them. So that’s my experience anyway, especially sɛ me kɔ me yɛ me clinicals a me kɔ maternity side no [when I go to do my clinicals at the maternity side], that was the only part where I felt some way, it’s like ah what is this?*

Another nurse also recounted that:

*…imagine sɛ wo colleague ɔyɛ general nurse anaa midwife, na especially yɛ wɔ school na yɛ service ne clinicals ne ade, na ɔmo kɔ hyia a whole eyi Director of nursing service, oh wo firi school bɛn, oh me firi maybe Ankaful Psychiatric Nurses Training College, eeei mo aba ha, have you seen? they say eeii like they are kind of surprised. Eei mo aba ha, mo saa nkorɔfo yi deɛ, you know, so they brand us as the way they brand our patients eii abɔdam, abɔdam and they will be calling you abɔdam you know…* [imagine that a colleague who is a general nurse or midwife and we go for national service and clinicals and we go to meet a whole Director of nursing service, and they ask which school you are coming from, and I said, ‘I’m from Ankaful Psychiatric Nurses Training College,’ he said ‘eei! You’ve come here. As for you these people.’ They say ‘eei’ like they are kind of surprised. So, they brand us as the way they brand our patients ‘eei mad people, mad people’…].

### Structural stigma and mental healthcare

Participants asserted that mental healthcare is neglected in Ghana. The government does not give enough recognition and support to mental healthcare. According to participants, mental health is of less concern to the government. They asserted that this situation is partly due to the perception that mentally ill patients have no potential or cannot become “normal” citizens that will add to the country’s economic development. Thus, spending state resources on their healthcare is loss to the state, as asserted by this female nurse:

*…we are the least to the government problems, we have been relegated to the background because like I told you from the beginning since Nkrumah, they have not built any psychiatry hospital. How many times they talk mental health is even appalling. Nobody is going to benefit; government will not get involved. Patients too are not working, do you understand? They do not get anything from them so why should they invest it.*

According to participants, most government officials or their immediate family members hardly utilize the mental healthcare facilities in the country, which also fuels the neglect. Because these government officials have the financial resources to travel to developed countries to find treatment for their mental health issues or that of their family, they care less about the vulnerable [mentally ill] people in society:

*…the government is just not interested in um the welfare of the people after all when they, when their family members get sick like psychological issues, they apply them to the best of care, they can apply them to maybe either UK, South Africa or anywhere for the best of care but the ordinary Ghanaian we do not have, none we do not have the government psychiatry hospital or a state hospital in central Ghana or in the northern part of the country.*

Another nurse also explained that:

*Ankaful sei Kwame Nkrumah mmerɛ so na ɔmo si Ankaful hospital, there have not been any renovation, benevolent people na ɛba hɔ na ɔmo abɛ renovati. No Ghanaian government has come na wo bɛ ka sɛ okay Ankaful kraa sei a me ba bɛ ti me ho abɛ yɛ sei anaa sei. Ɛka wakoma a institutions ɛne um others na ɔmo abɛ donate nneɛma ɛne renovation nkakra kakra. Government ɔmo nhwɛ mental illness.* [Ankaful like this, it was during the era Kwame Nkrumah that they built it. There has not been any renovation, it is benevolent people that come to renovate it. No Ghanaian government has come and said let us go and renovate it. When it touches an individual or a private institution’s heart, then they will come and donate. The government does not regard mental illness.]

### Effects of stigma on infrastructure and service provision

The nurses asserted that they lack basic equipment and resources for delivery of services, and this situation has led them to improvise mostly in their service delivery, which they think is not the best for their patients’ full recovery. A nurse lamented that:

*One of the challenges that we face is lack of equipment to help us do our work. We do not have most of the things too, so most of the things we do is just improvising, you know, and it worries a lot. […] there is no equipment that will be provided or there are no infrastructures put in place to help in delivering your job, so it makes work very difficult. […] we are not motivated enough, we do not have much equipment in delivering our service, so those are the major challenges that we face.*

Participants asserted that another major challenge facing mental healthcare in the country is the shortage of medication for treatment. They emphasized “we have instances that we may have shortage of medications for our client. […] medications have been basis of our challenges.”

Participants explained that though there are community mental health units in most community health centers and primary healthcare facilities, resources for mental health service provision are not readily available to aid in treatment and care at these facilities. Though mental healthcare services have been extended to other primary healthcare sectors, participants asserted that those mental health units still lack the necessary resources like medication and specialized professionals:

*…the community psychiatric nurses are also trained but they are […] not getting any office or any place to work, [and] these people instead of practicing mental health, they are then turned into general practitioners. So, instead of them to do follow ups on mental health cases and stuff, they do not do that, so people are always having problems over there.*

Nurses explained the importance of building psychiatric hospitals in other parts of the country, especially in the northern and middle parts, asserting that:

*…it will be important that we bring some [psychiatric hospitals] in the northern part of the country and some in the middle belt of the country whereby not everything will be centered in the southern part to put that kind of pressure. And it makes those in the northern part and the middle part left out, they feel neglected so it’s a big challenge to us so policy makers should look at these things as well.*

### Effects of stigma on human resources

According to the nurses, the government’s neglect toward mental healthcare affects the human resources available to provide mental healthcare services in the country. Participants asserted that there are not enough psychiatric nurse training colleges in the country to train mental health nurses. One nurse stated that:

*mental health nurses are not many. There are only three psychiatric hospitals in Ghana and only two psychiatric hospitals train psychiatric nurses. So, you do your calculation. We have so many general nurses all over the region but only two, two psychiatric hospitals pɛ [only]. And these two psychiatric nurses, nipa no nfiri Ankaful training a ofiri Pantang training [if the person is not from Ankaful training, then she is from Pantang training].*

The nurses stated that there are few medical students who want to specialize in psychiatry. To participants, this situation is due to the stigma and negative perceptions attached to mental illness. This causes a deficit in the human resources available to provide mental healthcare in the country. Nurses also talked about lack of specialized mental health professionals like child psychiatrists, psychologists, gerontic nurses, nutritionists, among others, that are essential to patients’ recovery. They explained that these specialized mental health professionals are needed at the hospital to support patients’ full recovery:

*I’m saying that the whole hospital does not have a psychologist. We do not have a psychologist and you cannot treat mental illness without a psychologist. […] we need people who are professionals okay. People who understand mental health and are willing to work and make it work.*

Nurses acknowledged that there is an active brain drain of mental health nurses in the country, which is very alarming. They explained that most psychiatric nurses are moving to other countries, especially the United Kingdom, to work as mental health nurses because of good salary and better equipment to aid their work. A nurse explained that:

*…nobody is doing anything so now the thing is just write your IELTS [International English Language Testing System], pass and then the agencies are already there. So, all you need to do is to apply to them sɛ wo passi [you have passed your] IELTS. Some even go to the extent of sponsoring you na wo ankasa akɔ kyerɛw, wo passi a ɔmo a bɛ yɛ processes no ama wo [for you to go and write and when you pass, they do all the processes for you] because they need the personnel there and they have realized sԑ [that] mental health nurses from Ghana, from like West Africa, ɔmo ba hɔ no mo a, [when they come there], they perform extremely well. Um for instance UK sei wanhu sɛ yɛ no ɔmo English no ɛyɛ eyi nti [like this, you have seen that our English and theirs are similar so when they go there] […] they perform well, resources ne yԑ [are] available ԑna [and] incentives nso wɔ hɔ mo [are available], but here biribi saa nni hɔ [there is nothing like that].*

## Discussion

The study explored interpersonal and structural stigmas surrounding mental illness and their impact on mental healthcare in Ghana from the perspectives of psychiatric nurses from the Ankaful Psychiatric Hospital, Ghana. The study identified that social and structural stigmas associated with mental illness seriously impact the work of psychiatric nurses and institutional psychiatric care in Ghana. The findings illustrate that while social stigma makes psychiatric nursing a difficult career choice for many aspiring nurses and entrenches power struggle and structural limitations within the nursing field, structural stigma—resulting from governmental neglect and lack of material resource provision—presents difficulties for nurses and other mental health professionals in the exercise of their care duties for their clients.

Stigma toward mental health and its influence toward mental healthcare career choice and retention is well documented in mental health literature (5, 29–36, 55, 56). In line with mental health literature in Ghana (7, 8, 29, 30), this study revealed that due to stigma attached to mental illness and the fear of aggression, most of the study participants did not want to do psychiatric nursing. Though they had interest in becoming nurses or working in the healthcare sector, psychiatry was not their initial career choice. According to the nurses, psychiatry is unattractive and unpopular in Ghana, which makes it a difficult choice as an area of specialty in the healthcare industry. This is evident in the literature on psychiatry as a career choice for aspiring health professionals (29, 30, 55, 56). Participants explained that the perception that people with mental illness are aggressive and unpredictable on their next move are widespread in Ghana, which partly produces and entrenches the stigma toward psychiatry as a career choice. Regardless, there are nurses who genuinely pursued psychiatry out of compassion for mentally ill vagrants. All the participants shared that they are appreciative of having the opportunity to pursue psychiatric nursing because it has reoriented their perception of mental illness and understanding of the complexities in human behaviors.

The nurses explained that psychiatry and psychiatric care workers are stigmatized due to their association with people with mental illness. This type of stigma has been widely termed as courtesy or associative stigma, which is defined as the stigma one receives due to their association or relation with a stigmatized group (37–39). Participants asserted that they are not being regarded as healthcare providers because they provide care for mentally ill people. It must be noted that this stigma toward psychiatry and psychiatric care providers is a result of the social stigma toward mental illness and people with mental illness (40). Participants explained that mental healthcare workers face similar social stigma as that of mentally ill persons in most Ghanaian societies. This stigma is further entrenched during encounters with their colleagues, primary healthcare providers such as midwives, general nurses, and doctors. According to participants, stigma from the primary healthcare providers is more discouraging than that of the society. This stigmatizing behaviors toward psychiatric nurses by primary care providers are doing something more than just societal reproach of association with mental illness or people with mental illness; it demonstrates power struggle and structural limitations within the nursing field (37, 41).

Link and Phelan establish that one reason behind stereotypes and stigma is to ensure the loss of status and power for the stigmatized group (37, 41). Stigma toward psychiatric nurses by other primary care nurses ensures that psychiatric nurses maintain the lower ranks in the nursing field in Ghana. Psychiatric nurses, according to participants, are not regarded during clinical encounters at the hospitals in their school days as being sidelined during major nursing programs and meetings when working as registered nurses. Though these stigmatizing behaviors are not overt to the public, they ensure that psychiatric nurses gain no power or are ascribed low status in the healthcare system in Ghana (41). Stigma from colleague healthcare providers, according to the psychiatric nurses, is detrimental to the development of psychiatry in Ghana as it stalls advocacy for mental healthcare in the country.

The study also identified that mental healthcare in Ghana faces structural stigma (37, 42–44). This, according to participants, results in governmental neglect and lack of material resource provision for mental healthcare in the country. According to participants, efforts to improve mental healthcare by successive governments after the 1970s are inadequate compared to other healthcare sectors within the country. Literature attests to this assertion of inadequate resource provision for mental healthcare in Ghana (45–52). Participants asserted that there is discrimination/stigma toward mental healthcare provision by governmental authorities and agencies, especially Ankaful Psychiatric Hospital, as it is located in a remote area. This form of stigma is mostly indirect; however, it has a negative impact on mental healthcare service provision. A ‘better’ service provision, according to the nurses, is largely based on the availability of material resources such as medications and infrastructure to support patients’ recovery. The lack of facilities and resources for mental healthcare provision is a major threat to the mental health wellbeing of citizens in the country (47, 53). According to participants, material resource provision for mental healthcare in Ghana is inadequate and limited, which makes them believe that mental healthcare has been “relegated” to the bottom of healthcare priorities in the country.

To participants, the government does not care about mental healthcare in the country, and it is a result of the societal stigma toward mental illness. This is supported by Coe’s assertion that “care is understood to take place through the provision of the necessities of life” [(54), p. 11]. Here, participants evaluated the government’s dearth in the provision of material resources at the hospital as lack of care toward people with mental illness and their caregivers. Care is understood to be dependent on the provider’s ability to provide both intimate relations and material resources. Materiality of care is as important as relational care, as Coe establishes in her work among Ghanaian transnational families. Where immigrant parents become good parents through the provision of material resources such as sending remittances to their children for their upkeep in Ghana (54). Analyzing the perspectives of participants, the government’s intimate/relational care is seen through the decentralization of mental healthcare services as enshrined in the new Mental Health Act 846. However, the government’s inability to provide adequate material resources to support care provision in these facilities undermines their relational care. This particular challenge (inadequate resource provision) was identified by Doku, Wusu-Takyi, and Awakame (48) in their paper on the implementation of the law, where they asserted an exercise of caution by the government in the implementation of the new law, which they believe can add to the already existing “perennial burdens” facing mental healthcare in Ghana, such as underfunding and understaffing.

Mental illness stigma does not only affect people with mental health issues but also caretakers and mental health service providers. Stigma toward psychiatric nurses and, by extension, the mental healthcare sector is largely due to the mental health conditions of their clients, which negatively impacts mental healthcare provision in Ghana. The psychiatric nurses asserted that stigmas from community members and other healthcare providers do not significantly impact their work as compared to structural stigma from the government. Though psychiatric nurses in Ghana care about their clients, the inadequate resources to aid their work have made them unenthusiastic in their delivery of care for clients. Relational care is influenced by the material conditions of service (54). As Coe’s work demonstrates, material resource provision is much appreciated in care practices, as it is seen as showing of concern and thinking of the wellbeing of the cared for. Material resource provision for mental healthcare is a demonstration of the government’s practice of care for the mentally ill. The ability of the government to provide the needed material resources to care for people with mental health issues in various parts of the country shows the government care about both the mentally ill patients and the mental health nurses/workers. This is because the provision of these resources will not only ensure that the best of care or treatment is provided for the patients, but the safety and wellbeing of the mental health workers are also catered for.

The stigma of mental illness, and by extension psychiatry, negatively influences career choice in psychiatry by medical students (55–57) and psychiatric nursing (29). Psychiatry as a profession is in itself not an enticing career choice for many because it does not bring enough income and is stigmatized. In Ghana, literature identifies that mental health service providers are stigmatized by society and other healthcare providers due to their profession and role in providing care for persons with mental illness (7, 8, 29). Literature identifies that the stigma of mental illness not only affects persons with mental health issues but extends to psychiatric care providers (30). Stigma, in addition to other socioeconomic factors, negatively affects the recruitment and retention of psychiatric care providers, especially in low- and middle-income countries (29, 30). Due to these stigmas, especially structural stigma, nursing career choice and retention in psychiatry has become a significant challenge in the country. Nurses are either leaving the country to work abroad or changing to other fields within the healthcare due to inadequate resources to aid their work as psychiatric nurses. This affects client health outcomes as there are inadequate resources (both human and non-human) to provide care at the psychiatric hospitals. Mental healthcare in Ghana significantly lacks quality, and the number of psychiatric nurses keep dwindling, especially in recent years, as there has not been recruitment of nurses into government-owned healthcare facilities for about 3 years.

## Conclusion

The article provides insights into the stigma experiences of psychiatric nurses and the impact of these stigmas on mental healthcare provision in Ghana. The study addresses a relevant gap in the literature and contributes to efforts to improve mental healthcare policymaking and advocacy. It contributes to research on mental health systems from provider perspectives by focusing on the experiences and perspectives of psychiatric nurses on mental illness stigma and psychiatric care provision in Ghana. The article adds to efforts of the global mental health movement to improve mental healthcare policymaking and advocacy in low- and middle-income countries. It also adds to the expansion of mental health literature from the perspectives of psychiatric nurses from low- and middle-income countries, specifically Sub-Saharan Africa. I call for further research using different methodologies to focus on the experiences of other psychiatric care providers to beef up the goals of the global mental health movement.

### Limitations/challenges

First, it is pertinent to recognize that this study represents the perspectives of psychiatric nurses from the Ankaful Psychiatric Hospital, Ghana. Patients were not involved in the study due to ethical issues that come with online interviews. Access to medical officers and other mental healthcare providers through referral was impossible, and the time for the research was limited due to institutional requirements. Hence, the decision to interview only mental health nurses since they were the people I could get in touch with. The study faced several challenges caused by the COVID-19 pandemic that hit the world in 2020, such as restrictions on traveling around the world. Due to these pandemic challenges, the initial research design changed to enable internet-based qualitative interviews. Seeking ethical clearance was another challenge that the project faced. It took more than 3 months for ethical approval to be given by the Northern Arizona University Institutional Review Board (NAU IRB), the Ghana Health Services Ethics Review Committee, and the Ankaful Psychiatric Hospital Research Ethics Review Committee.

The study’s small sample size limits its generalizability, but it can be replicated in other settings or populations with a similar focus. Future research is encouraged to include participants who otherwise would have been included if not for the COVID-19 pandemic challenges. In addition, as already stated, the study relies on remote interviews, which may limit rapport building and observation of non-verbal cues during interviews. I addressed this limitation by making the interviews more engaging and conversational, which allowed participants to share their stories with no reservations. As a Ghanaian, I adopted a bilingual approach when talking to participants. Ghana is a highly bilingual country, which is reflected in our conversations as we use both English and our local dialect. In my case and that of my participants, we spoke English and Twi interchangeably. Bilingualism in a Ghanaian conversation creates a more informal way of communication, and this offered participants the flexibility to express themselves and helped build rapport through a remote interview.

## Data Availability

The raw data supporting the conclusions of this article will be made available by the authors, without undue reservation.
